# Root Exudate-Induced Alterations in *Bacillus cereus* Cell Wall Contribute to Root Colonization and Plant Growth Promotion 

**DOI:** 10.1371/journal.pone.0078369

**Published:** 2013-10-24

**Authors:** Swarnalee Dutta, T. Swaroopa Rani, Appa Rao Podile

**Affiliations:** Department of Plant Sciences, University of Hyderabad, Hyderabad, Andhra Pradesh, India; Rockefeller University, United States of America

## Abstract

The outcome of an interaction between plant growth promoting rhizobacteria and plants may depend on the chemical composition of root exudates (REs). We report the colonization of tobacco, and not groundnut, roots by a non-rhizospheric Bacillus *cereus* (MTCC 430). There was a differential alteration in the cell wall components of *B. cereus* in response to the REs from tobacco and groundnut. Attenuated total reflectance infrared spectroscopy revealed a split in amide I region of *B. cereus* cells exposed to tobacco-root exudates (TRE), compared to those exposed to groundnut-root exudates (GRE). In addition, changes in exopolysaccharides and lipid-packing were observed in *B. cereus* grown in TRE-amended minimal media that were not detectable in GRE-amended media. Cell-wall proteome analyses revealed upregulation of oxidative stress-related alkyl hydroperoxide reductase, and DNA-protecting protein chain (Dlp-2), in response to GRE and TRE, respectively. Metabolism-related enzymes like 2-amino-3-ketobutyrate coenzyme A ligase and 2-methylcitrate dehydratase and a 60 kDa chaperonin were up-regulated in response to TRE and GRE. In response to *B. cereus*, the plant roots altered their exudate-chemodiversity with respect to carbohydrates, organic acids, alkanes, and polyols. TRE-induced changes in surface components of *B. cereus* may contribute to successful root colonization and subsequent plant growth promotion.

## Introduction

Plant roots influence rhizospheric microenvironment through root exudates (REs). For instance, carbon-rich photosynthates in REs facilitate sustenance of higher microbial populations and activity in rhizosphere, compared to bulk soil. Selection of bacteria in the root zone depends on niche utilization as well as rhizospheric-processes that reciprocate between the host plant and bacteria. Specifically, RE-mediated early interactions between the plant and bacteria determine the fate of plant-microbe association. Presence of various ions, free oxygen, water, enzymes, mucilage and a diverse array of primary and secondary metabolites in the REs might deter one organism and attract the other. Alternatively, two very different organisms may get attracted with differing consequences to the plant. Differences in chemotactic responses of microbes towards amino acids, sugars and organic acids in the REs influence their ability to colonize roots [1]. Root nodulating-rhizobia sense flavonoids and betaines secreted by the host root, and respond by expressing *nod* genes [2,3]. Components of REs, like acetosyringone, induce the expression of virulence genes in *Agrobacterium* [4]. The REs, therefore, play an important role in plant-microbe interactions.

Flagella, fimbriae and pili enable the bacteria to attach to plant surface [5]. Bacterial flagellins play a crucial role for the beneficial bacteria in recognizing host and non-host plants [6,7]. Cell surface polysaccharides are involved in the establishment of a symbiotic relationship between legumes and rhizobia [8]. Bacterial major outer membrane protein (MOMP) also plays an important role in early host recognition. The MOMP was involved in root adsorption and bacterial-cell aggregation [9]. The MOMP of *Azospirillum brasilense* exhibited stronger adhesion in response to the extracts of cereals than extracts of legumes or tomato [9]. Similar to flagella and MOMP, the bacterial cell wall (CW) components also play a crucial role in mediating plant-rhizobacterial interaction. Protein profiling of *Paenibacillus polymyxa*, isolated from barley-rhizosphere, showed quantitative changes in both cytosolic and extracellular proteins in presence and absence of barley [10]. Soybean growth promoting *Bacillus amyloliquefaciens* and its UV-mutant with reduced ability to promote growth displayed differential expression of extracellular proteins [11]. Identification of changes in bacterial CW proteins (CWPs) in response to REs may, therefore, provide information on the bacterial proteins that facilitate root colonization.

Characterization of the bacterial response to REs at the cell surface requires extraction, fractionation, and analysis of cell surface components. Non-invasive vibrational spectroscopy, including fourier transform infrared (FT-IR) and attenuated total reflectance infrared (ATR-IR), was used as alternative to study the intact bacterial cells [12-14] to obtain information regarding the functional group chemistry of bacterial cell surfaces. 

Soil bacteria from rhizosphere, non-rhizospheric zones, phylloplane etc., promote plant growth suggesting that the interaction of the bacterial strains with plants was not dependant on their habitat. Further, a few strains of plant growth promoting rhizobacteria (PGPR) promoted growth of several crop plants, while others enhanced the growth of one or a few crop plants. To understand the host and bacterial factors that contribute to plant-PGPR association, we have adopted a new approach. We have selected five bacterial strains (*Bacillus cereus*, *B. subtilis*, *Paenibacillus elgii*, *Serratia marcescens*, and *Stenotrophomonas maltophilia*) that were not isolated from the rhizosphere and studied their effect on four crop plants (tobacco, groundnut, tomato, and pigeon pea). Some of these bacterial isolates were reported as PGPR, suggesting that the plant growth promoting ability was not limited to the bacteria isolated from rhizosphere [12,15]. Subsequently, *B. cereus* that promoted growth of tobacco, and not of groundnut, was selected to study the changes in cell-wall components through ATR-IR (intact cells) and 2-DE analysis of CWPs in response to the REs of tobacco and groundnut. Further, chemical composition of tobacco as well as groundnut REs from plants treated with or without *B. cereus*, was evaluated. We report that RE-induced changes in *B. cereus* cell surface have implications in root colonization and consequent growth promotion.

## Materials and Methods

### Selection of bacterial strain

Five bacterial isolates were screened for their growth promoting ability on tobacco under *in vitro* conditions. *B. subtilis* (MTCC9447) and *B. cereus* (MTCC 430) were from Microbial Type Culture Collection, Institute of Microbial Technology, Chandigarh, India. *P. elgii* [12] was isolated from chitin/chitosan-rich soils of a mushroom production firm, S. M. Agritech Pvt. Ltd., Hyderabad, India. *S. marcescens* [16] and *S. maltophilia* (K279A) are from our culture collection in Department of Plant Sciences, University of Hyderabad. *B. cereus* (MTCC 430) that promoted the growth of tobacco, was used to bacterize the seeds of four different plants including groundnut, tobacco, tomato and pigeonpea. 

### Growth promotion studies *in vitro*

Tobacco and tomato seeds were surface sterilized by placing them in 1% sodium hypochlorite solution (v/v) for 30 seconds followed by rinsing three times with sterile distilled water. Groundnut and pigeonpea seeds were surface sterilized with 0.02% (w/v) mercuric chloride and washed four or five times with sterile distilled water. Selected bacterial strains were grown overnight in 50 ml of Luria-Bertani (LB) broth. Cells were collected by centrifugation at 3,200 x *g* for 5 min. Sterile distilled water was added to the cell pellet to suspend bacterial cells. Surface sterilized seeds were steeped in the bacterial suspension for 30 min and dried for 1 h to achieve 1 x 10^7^ cfu/seed. Seeds treated with sterile distilled water served as control. Treated seeds were placed in the Murashige and Skoog’s (MS) medium. The shoot height, root length, fresh and dry weight of the plants was recorded after 30 days of growth. Root colonization by bacteria on tobacco and groundnut was assessed through serial dilution after 10, 20, 30 and 40 days for tobacco and 5, 10, 15 and 20 days for groundnut. 

The data on growth promotion from triplicates, in three independent experiments, was analyzed by Duncan’s multiple range test (DMRT) and CD value was calculated at p_0.05_. Significant difference between the treatments was designated by different letters. The effect of *B. cereus* on growth of selected crop plants was analysed by students’ t-test for individual parameter against control for each crop. Percent increase over control was calculated and plotted, where required. 

### Preparation of REs

The REs were collected according to Slavov et al. [17]. Tobacco and groundnut plants were grown on MS media for 20 and 5 days, respectively. After careful washing of the roots, the seedlings were transferred to flasks containing 200 ml of sterile distilled water and kept for 2 days. Sterile distilled water, without nutrients, was added as required to maintain a constant volume. The aqueous media containing the compounds released by the roots were collected and filter sterilized (0.22 µM, Millipore, USA). The volume was adjusted to 100 ml/g fresh root weight. The REs were kept at 4°C until used. Tobacco and groundnut root exudates were referred to as TRE and GRE, respectively.

### GC-MS analyses of REs

TRE and GRE were extracted in ethanol as reported by Nagahashi and Douds [18] for a detailed analysis. The REs were lyophilized, suspended in 80% ethanol and kept on ice for 2 h. After centrifugation at 8,200 x *g* for 5 min at 4°C, the supernatant was separated and concentrated. The dried supernatant (crude exudate) was dissolved in 70% ethanol and analyzed for metabolites (sugars, organic acids, alkanes, polyols, *etc*.) using GC-MS according to Schliemann et al. [19].

Ethanolic extracts (100µl aliquots) were derivatized after reducing to dryness in glass injection vials with 10µl N-methyl- N-(trimethylsilyl)-trifluoroacetamide for 30 min at 70°C and diluted with 90µl of hexane prior to injection. For derivatization of MeOH-soluble compounds, 10µl aliquots were first derivatized with 10µl methoxyamine hydrochloride (MOA, 20 mg ml^-1^ pyridine) for 90 min at 30°C, reduced to dryness and then treated as described for the hexane extracts. GC-MS measurements were obtained with Agilent 6890 series gas chromatograph equipped with an autosampler 7683 series. The following conditions were used: EI-voltage 70 eV; source temp. 240°C; column Rtx-5MS w/IntegraGuard (Restek GmbH, Bad Homburg, Germany), 30 m x 0.25 mm i.d., 0.25µm film thickness column (Agilent, Folsom, USA); carrier gas helium at constant flow of 1 ml min^-1^; temperature. program: 50°C (2 min), 50–260°C (6° min^-1^), 260°C (3 min), 260–300°C(10° min^-1^), 300°C (6 min); injection temperature: 240°C, splitless injection, mass range of m/z 40 to m/z 800. Data acquisition and evaluation run were with Xcalibur 1.4.1. Quantification of a selected set of metabolites was based on the measurements of reference compounds (separate and in mixtures) using characteristic fragment ions.

### Cell surface analysis for intact cells of *B. cereus* using ATR-IR spectroscopy

Bacterial samples were prepared according to Kamnev et al. [20]. *B. cereus* was grown to an OD_600_ of 0.5-0.6 in LB, minimal media (MM) and MM-amended with TRE/GRE (1:1, v/v). Cells were separated by centrifugation at 8,200 x *g* for 8 min at 4°C, washed three times with 0.85% NaCl solution, and finally washed with double distilled water. The cells were dried in air at 50°C for 8 h. The spectra for the air-dried bacterial cell pellet were recorded with a total of 64 scans at a resolution of 4 cm^-1^ in the transmission mode (mid-infrared region, 4000–400 cm^-1^) using a Nicolet 5700 FTIR spectrometer. Normalization and analysis of spectral data were done using Origin 6.1 software (OriginLab Corporation, Northhampton, MA, USA) and Speckwin32 software (Version 1.71.4, Jena, Germany). 

### Isolation of CWPs from *B. cereus*

The CWPs were isolated from *B. cereus* according to Cole et al. [21] with biological triplicates. Cells from 12-16 h old culture of *B. cereus* grown in LB, MM, MM+TRE and MM+GRE were harvested by centrifugation at 7,560 x *g* for 20 min at 4°C. Supernatant was discarded and pellet was kept on ice for 5 min. The pellet was washed twice with 5ml of chilled TE buffer containing 1mM PMSF. The pellet was resuspended in 1.15ml of ice-cold mutanolysin mix [1 ml TE-Sucrose (TES) buffer, 100 μl lysozyme (100 mg/ml in TES), 50 μl mutanolysin (5,000 U/ml in 0.1 M K_2_HPO_4_, pH 6.2)], prepared freshly before use and placed on ice. The whole suspension was transferred to a sterile microcentrifuge tube and incubated for 2 h at 37°C with shaking (220*g*). After incubation, the mixture was centrifuged at 14,000 x *g* for 5 min at 28°C. The supernatant (solubilized cell wall-associated protein fraction) was collected and dialysed with 6 changes (every 2 h) against double distilled water in a dialysis membrane from Himedia, India with 4 kDa cut-off. Dialysed proteins were acetone precipitated (1:4 v/v). The protein concentration was determined using amido black method and resolved on SDS-PAGE. The purity of CWP fraction was checked for lactate dehydrogenase (LDH) assay, a cytosolic marker enzyme, according to Bergmeyer and Bernt [22].

### 2DE analysis of differentially expressed CWP

Aliquots of 500 µg CWP were rehydrated on immobilized pH gradient (IPG) strips of 18 cm length and pH of 4-7 linear gradient (Amersham, GE Healthcare) for 12 h at 50 V with rehydration solution [8 M urea, 2 M thiourea, 4% CHAPS, 50 mM dithiothreitol (DTT), 0.2% IPG buffer pH range 4-7 and 0.004% bromophenol blue] to a final volume of 320 µL. Rehydration and isoelectric focusing (IEF) were carried out in Ettan IPGphor II (GE Healthcare) at 20°C using the program: 500 V for 30 min, gradually increasing to 500-10000 V for 3 h and then a step voltage up to 60000 Vh. After IEF, strips were equilibrated twice for 20 min with gentle rocking at 25 ± 2°C in equilibration buffers. The first equilibration was done in a solution containing 6 M urea, 50 mM Tris-HCl buffer (pH 8.8), 30% (w/v) glycerol, 2% (w/v) SDS and 2% DTT. In the second equilibration buffer, 2.5% (w/v) iodoacetamide was used instead of DTT. The proteins were separated in the second dimension SDS-PAGE (12.5% vertical polyacrylamide slab gels) at 4 mA gel^-1^ for 1 h and kept overnight at 10 mA gel^-1^, using an EttanDalt6 chamber (Amersham, GE Healthcare). The gels were stained with modified colloidal coomassie G-250 staining [23]. Protein patterns in the gels were recorded as digitized images using a calibrated densitometric scanner (Amersham, GE Healthcare). The gels were analyzed (normalization, spot matching, expression analyses and statistics) using Image Master 2-D Platinum image analysis software (Amersham, GE Healthcare). Gels with scatter plot correlation coefficient > 0.8 were further analysed for differentially expressed CWPs. 

Protein spots in the gels of CWP from *B. cereus* grown in MM+TRE and MM+GRE were compared with the protein spots on the master gel (CWP of *B. cereus* grown in MM). Differentially expressed proteins, between *B. cereus* grown in MM+TRE and MM+GRE, were compared against MM+TRE as master gel. Interclass report was calculated based on spot volume percentage and sample *verses* control ratios and compared. Protein spots with ratio >1.5 were considered as up-regulated and < 0.5 were considered as down-regulated. Student’s t-test was performed to identify statistically significant (p<0.05) differentially expressed proteins. Selected protein spots were digested with trypsin and identified through MALDI-TOF mass spectral data analysis using MASCOT program (http://www.matrixscience.com) employing Biotools software (Bruker Daltonics). The protein identity was accepted only if the MASCOT probability was at significant threshfold level (p<0.05) with at least two peptides matching.

## Results

### Selection of bacterial strain: seed bacterization and root colonization

**Figure 1:**
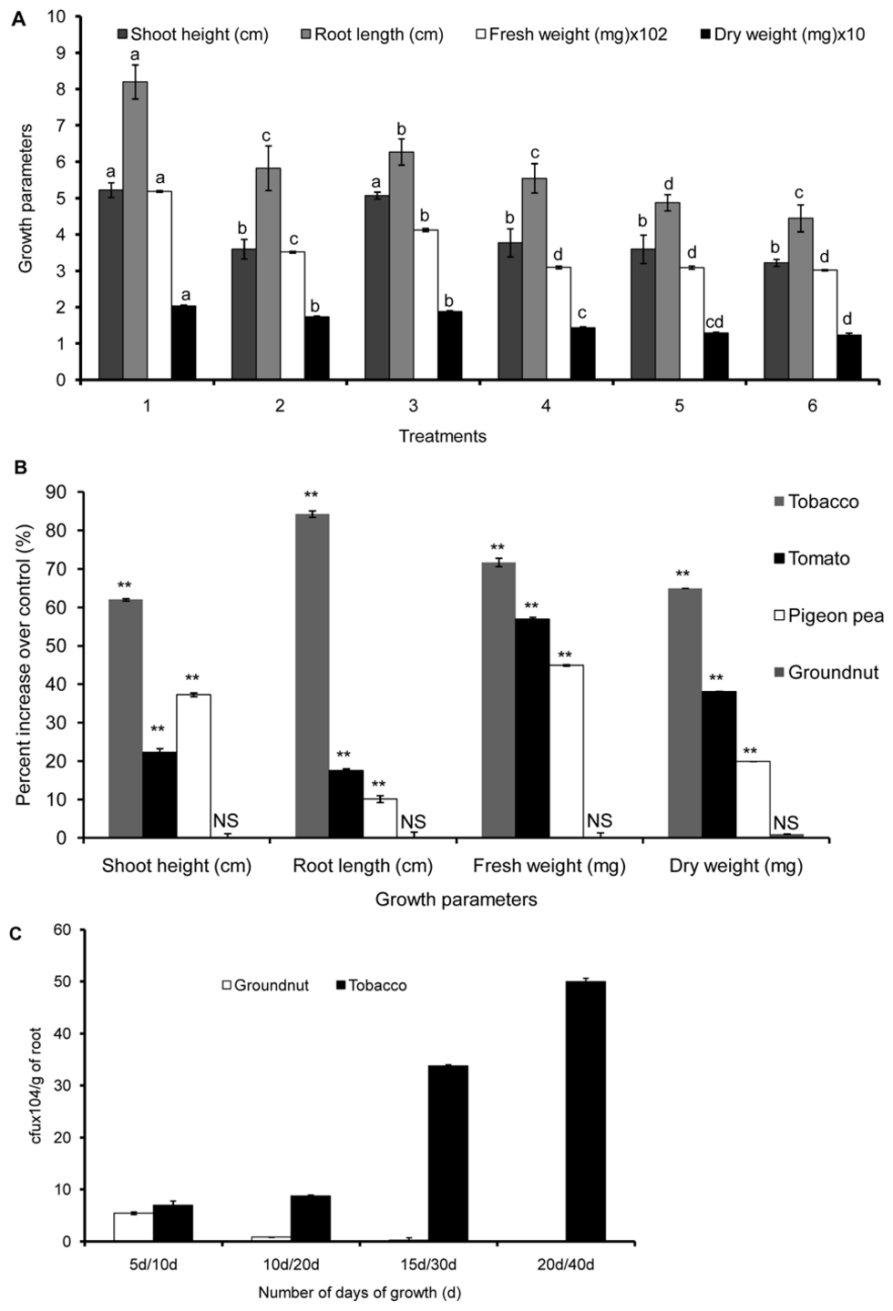
Effect of non-rhizospheric bacterial strains on growth of crop plants. Seeds bacterized with respective bacterial strains (approximately1x10^7^cfu/seed, unless otherwise mentioned) were grown *in* *vitro* in MS medium. After 30 days of growth, shoot height and root length were measured in centimeters, while fresh weight and dry weight of entire plant were measured in milligrams after 30 days of growth. Data represent the mean of the three independent experiments. The vertical line indicates standard error. (A) Effect of five different bacterial strains on growth of tobacco. Treatments included 1. *Bacillus* *cereus*, 2. *B. subtilis*, 3. *Paenibacillus* *elgii*, 4. *Stenotrophomonas* *maltophilia*, 5. *Serratia* *marcescens*, and 6. Control, (n=20). Different letters on each bar represent values that were significantly different (p_0.05_). (B) Effect of *B. cereus* on growth of tobacco, tomato, pigeon pea and groundnut (n=24). Data represents percent increase over control. (C) Colonization of *B. cereus* on tobacco and groundnut roots. Number of days (d) for tobacco: 10, 20, 30 and 40 days of growth and for groundnut: 5, 10, 15 and 20 days of growth (n=20). Students’ t-test of each growth parameter against control for each crop was performed. ** indicate statistically significant at p<0.01, NS =indicate not significant.

Seed bacterization with five bacterial strains improved the growth of tobacco when compared to the non-bacterized control. *B. cereus*-treated plants showed maximum increase in growth followed by *P. elgii* (Figure 1A). Seed bacterization of four different plants with *B. cereus* increased shoot height, root length, fresh and dry weight of tomato and pigeon pea but the increase in growth of tobacco was the highest (Figure 1B). *B. cereus* efficiently colonized the tobacco roots with a significant increase in the population by 20 days that sustained up to 40 days. In groundnut, *B. cereus* colonized the roots only up to 5 days, and the rhizoplane population decreased to undetectable level in the next 15 days (Figure 1C). We have, therefore, selected tobacco as responding (host) and groundnut as non-responding (non-host) plants for *B. cereus*, and studied the influence of TRE *vs*.GRE on the cell surface components of *B. cereus*. In groundnut, the highest population of *B. cereus* was detected on roots at 5 days of growth, and at 20 days on tobacco roots. For further studies, TRE and GRE were prepared at 20 days and 5 days, respectively.

### Changes in REs profile in response to root colonization by *B. cereus*

GC-MS analyses of REs collected from groundnut and tobacco plants showed an alteration in composition of organic acids, carbohydrates, polyols, alkanes and benzene derivatives (Table 1 & 2) in response to seed bacterization/root colonization by *B. cereus*. 

**Table 1:** Metabolites in the root exudates of groundnut at 5 days of growth of bacterized (*Bacillus cereus*) and non-bacterized seedlings.

**Sl. No.**	**Name of the compound^a^**		***m/z* ratio**	**R_t_(min)**	**Non-bacterized**	**Bacterized**
**Organic acids**						
1.	2-Propenoic acid		131	23.0	-	+
2.	Acrylic acid		55	25.8	-	+
3.	Butanoic acid		73	21.8	-	+
4.	Cis, 6-octadecenoic acid		73	30.9	-	+
5.	D-threo-pentonic acid		73	26.3	-	+
6.	Isobutanoic acid		71	19.5	-	+
7.	Nonanoic acid		73	19.1	-	+
8.	Palmitic acid		73	34.6	-	+
9.	Pentanoic acid		73	16.4	-	+
10.	Pentenoic acid		73	10.4	-	+
11.	Decanoic acid		73	26.1	+	-
12.	Ethanimidic acid		147	10.3	+	-
13.	Gluconic acid		73	22.4	+	-
14.	Glyceryl tridodecanoate		43	43.8	+	-
15.	Hexanedioic acid		261	35.6	+	-
16.	Myristic acid		73	33.5	+	-
17.	Trans-9-octadecenoic acid		73	34.6	+	-
18.	Xylonic acid		73	24.7	+	-
**Sugars/Polysaccharides**						
1.		2-Deoxy-galactopyranose	73	32.7	-	+
2.		Arabinofuranose	217	27.4	-	+
3.		β-mannopyranoside	73	28.9	-	+
4.		Glucuronolactone	73	24.7	-	+
5.		Ribofuranose	217	25.2	-	+
6.		Sorbose	73	28.7	-	+
7.		2(3H)-furanone	73	20.8	+	-
8.		3-methyl-1,4,6,7-tetrahydro-pyrazolo[3,4-C]pyridin-5-one	42	13.1	+	-
9.		β-D-galactopyranoside	73	29.3	+	-
10.		β-fucopyranose	73	28.9	+	-
11.		D-arabinose	73	25.7	+	-
12.		D-erythro-pentopyranose	73	27.3	+	-
13.		D-erythrotetrofuranose	73	21.9	+	-
14.		Dithiothreitol	73	15.3	+	-
15.		D-turanose	73	40.9	+	-
16.		Fructose	73	28.7	+	-
17.		Pyrimidine	255	20.2	+	-
18.		Ribose	73	31.3	+	-
19.		Uridine	73	38.1	+	-
20.		Xylopyranose	73	23.6	+	-
21.		Xylose	73	26.5	+	-
**Polyols**						
1.	1,3-pentanediol		73	19.9	-	+
2.	2-methyl-1,2-butanediol		73	23.9	-	+
3.	2-methyl-1,3-propanediol		117	12.1	-	+
4.	Glycerol		73	17.3	-	+
5.	1-decanol		57	22.0	+	-
6.	1-heptanol		69	30.4	+	-
7.	2-ethyl-1-dodecanol		57	33.3	+	-
8.	Rhamnitol		117	22.0	+	-
9.	Ribitol		73	14.0	+	-
**Alkanes**						
1.	Butane		73	25.7	-	+
2.	Tetracosane		57	30.6	-	+
3.	n-docosane		57	22.9	-	+
4.	Tridecane		57	17.7	+	-
5.	n-dodecane		57	15.4	+	-
6.	Tetracontane		57	44.6	+	-
7.	1,2-bis(trimethylsiloxy)ethane		147	11.1	+	-
8.	2-ethyl-1,3-propane		73	23.7	+	-
9.	4-ethyloctane		57	10.8	+	-
**Benzene derivatives**						
1.	1,2,4-trimethylbenzene		105	11.4	+	-

a based on MS of reference compounds

‘+ ’ present; ‘- ’ absent

**Table 2:** Metabolites in the root exudates of tobacco at 20 days of growth of bacterized (*Bacillus cereus*) and non-bacterized seedlings.

**Sl. No.**	**Name of the compound^a^**	***m/z* ratio**	**R_t_ (min)**	**Non-bacterized**	**Bacterized**
**Organic acids**					
1.	2,3,4-trihydroxybutyric acid	73	23.3	-	+
2.	2-furylglycolic acid	73	15.8	-	+
3.	2-hydroxyheptanoic acid	73	20.6	-	+
4.	2-keto-D-gluconic acid	73	28.5	-	+
5.	Dodecanedioic acid	73	44.6	-	+
6.	Dodecanoic acid	73	22.7	-	+
7.	Mannonic acid	73	32.7	-	+
8.	Octadecanoic acid	117	34.9	-	+
9.	Octanoic acid	73	10.9	-	+
10.	Oleic acid	73	36.7	-	+
11.	2-trimethylsilyloxyheptanoic acid	73	20.6	+	-
12.	3-octeneoic acid	73	21.7	+	-
13.	Acetic acid	73	12.9	+	-
14.	Arabinonic acid	73	22.2	+	-
15.	Decanoic acid	73	43.0	+	-
16.	D-ribonic acid	73	25.1	+	-
17.	Ethyltrimethylsilyl dipropylmalonate	73	39.7	+	-
18.	Pentanedioic acid	73	36.2	+	-
19.	Pentanoic acid	127	27.1	+	-
20.	Tetronic acid	73	23.4	+	-
**Sugars**					
1.	1H-indole, 2,3,5-trimethyl-1-(trimethylsilyl)	231	36.0	-	+
2.	2-(2-furyl)pyridine	73	4.6	-	+
3.	5-[e,E-[4-[piperidino]-1,3-butadienyl]-2,6-dimethyl-1,2,4-triazine	192	7.7	-	+
4.	5-Ketofructose	204	46.9	-	+
5.	α-D-glucopyranoside	73	35.7	-	+
6.	β-D-galactofuranoside	73	32.1	-	+
7.	β-L-galactopyranose	73	26.3	-	+
8.	β-L-galactopyranose	204	35.1	-	+
9.	Methyl-keton	73	9.7	-	+
10.	Sorbopyranose	73	28.6	-	+
11.	α-D-mannofuranoside	129	27.8	+	-
12.	α-DL-arabinofuranoside	217	27.5	+	-
13.	Arabino-1,5-lactone	73	24.8	+	-
14.	Arabinofuranose	217	41.5	+	-
15.	D-fructose	73	29.6	+	-
16.	D-ribofuranose	217	28.5	+	-
17.	D-xylofuranose	217	28.0	+	-
18.	Glucofuranoside	145	27.3	+	-
19.	Pyridine	84	13.6	+	-
**Polyols**					
1.	2-methyl-1,3-propanediol	117	11.8	-	+
2.	Inositol	73	33.1	-	+
3.	Trimethylsilyl ether of glycerol	73	11.6	-	+
4.	{2,2-Dimethyl-5-[2-(2-trimethylsilylethoxymethoxy)propyl][1,3]dioxolan-4-yl}methanol	73	26.6	+	-
5.	Glycerol	73	14.0	+	-
6.	Valerenol	73	30.1	+	-
**Alkanes**					
1.	Butane	117	11.6	-	+
2.	Eicosane	57	36.1	-	+
3.	n-heptadecylcyclohexane	82	36.9	-	+
4.	Octacosane	57	39.0	-	+
5.	2-ethoxy-ethane	73	16.5	+	-
6.	2-ethyl-propane	73	23.8	+	-
7.	Hexadecane	328	36.9	+	-
8.	n-docosane	57	26.6	+	-
**Benzene derivatives**					
1.	1,2-dichloro-4-phenoxy-benzene	238	8.7	-	+
2.	2-hydroxybenzoic acid	73	18.8	-	+
3.	Diethyl phthalate	149	21.0	-	+
4.	2H-cyclopropa[g]benzofuran	73	29.9	+	-
5.	4-Acetyl-2-methoxy-benzene	193	21.9	+	-

a based on MS of reference compounds

‘+ ’ present; ‘- ’ absent

#### Organic acids

At 5 days of growth, 19 organic acids were common in the REs of both bacterized and non-bacterized groundnut seedlings. Ten organic acids were detected only in *B. cereus*-treated GRE and 8 others were detected in REs of non-bacterized seedlings (Table 1). In the TRE obtained from 20 days-old tobacco seedlings, 6 among the 26 organic acids detected were common in both bacterized and non-bacterized REs. Eleven organic acids were present only in TRE of bacterized seedlings, whereas 9 were exclusive to the TRE from non-bacterized tobacco (Table 2). There was an increase in the variety of organic acids exuded by *B. cereus* colonized tobacco plants.

Hexadecanoic acid, tetradecanoic acid, decanoic acid and propanoic acid were the organic acids detected commonly in TRE and GRE. The presence or absence of bacteria from groundnut roots did not alter the exudation of acetic acid and ocatadecanoic acid. But, acetic acid was not detected in TRE of bacterized seedlings, whereas, octadecanoic acid was present in the REs of bacterized seedlings.

#### Carbohydrates

Of the 32 carbohydrate-like compounds detected in GRE, 11 were common in the REs of bacterized and non-bacterized groundnut seedlings. Six carbohydrate-related compounds were detected exclusively in GRE of bacterized seedlings and 15 such compounds were detected in REs of non-bacterized groundnut seedlings (Table 1). There was a decrease in carbohydrates in GRE, subsequent to root colonization by *B. cereus*. Of the 22 carbohydrate-like compounds detected in TRE, only 3 were common in the REs from bacterized and non-bacterized tobacco seedlings, whereas 11 were detected in REs of bacterized tobacco seedlings and 8 in the REs of non-bacterized seedlings (Table 2). Contrary to the observation in GRE, *B. cereus* colonized tobacco plants showed an increase in sugar compounds in REs as compared to non-bacterized REs.

#### Polyols, alkanes and benzene derivatives

The profile of polyols detected in REs was different for tobacco and groundnut except for glycerol and inositol (Table 1 & 2). The alkane n-docosane was exuded in the TRE of non-bacterized seedlings (Table 2) but the same was present in GRE of bacterized seedlings (Table 1). Benzene derivatives were more in number in TRE as compared to GRE (Table 1 & 2).

### Changes in cell surface components of *B. cereus* in presence of TRE and GRE

**Figure 2:**
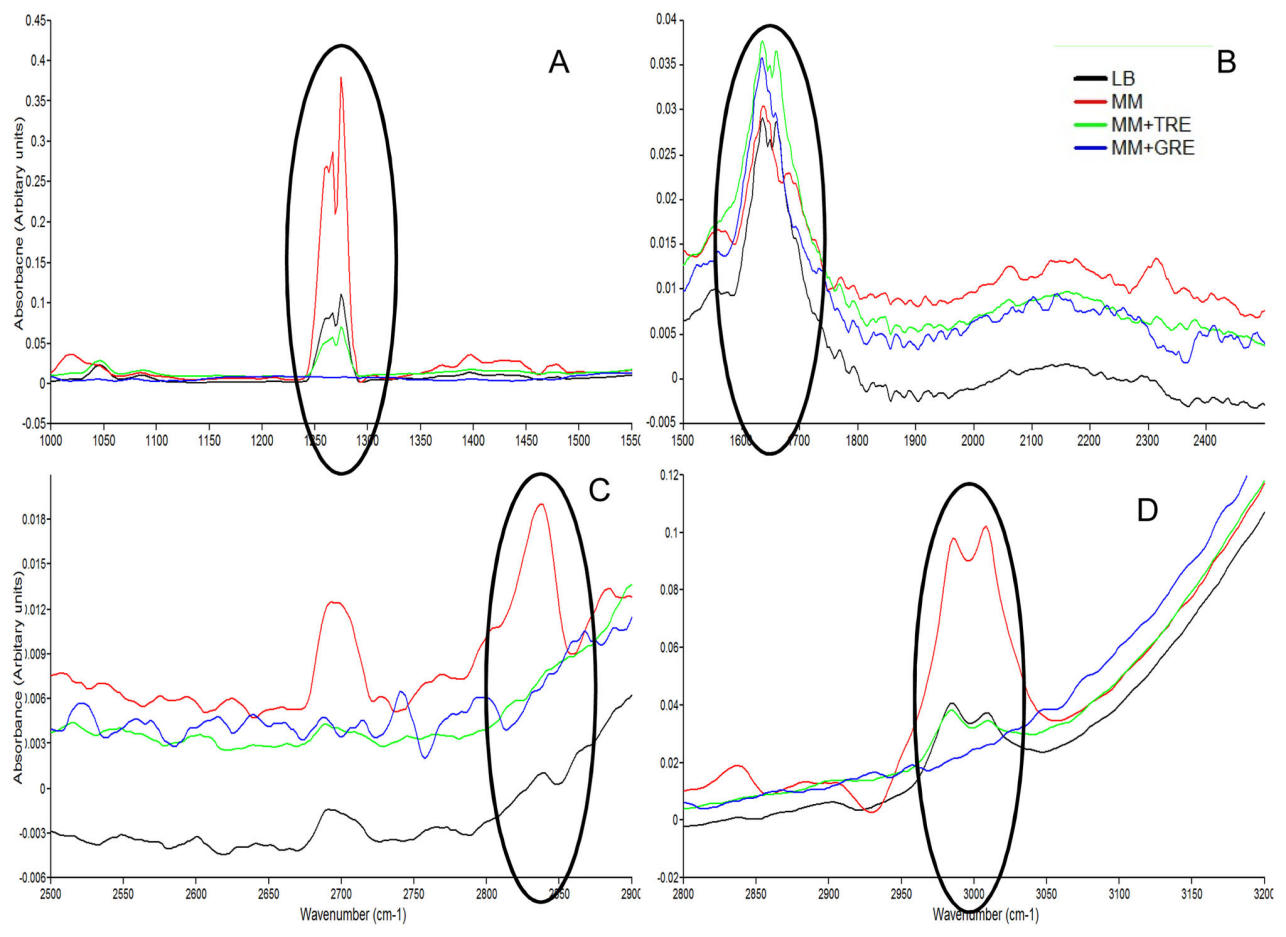
ATR-IR spectra of *B. cereus* grown in different media. (A) 1000–1500 cm^-1^ (B) 1500-2500 cm^-1^ (C) 2500–2900 cm^-1^ & (D) 2900-4000 cm^-1^ region of *B. cereus* grown up to OD _600_ = 0.5–0.6 in different media *viz*. LB, MM, and MM with TRE or GRE, and the intact cells were analysed. Spectra of the air-dried *B. cereus* cell pellet were recorded with a total of 64 scans at a resolution of 4 cm^-1^ in the transmission mode (mid-infrared region, 4000–400 cm^-1^) using a Nicolet 5700 FTIR spectrometer. Circles highlight the spectral areas with alteration in bands for bacteria grown in different media.

*B. cereus* was grown in LB, MM and MM-amended with TRE or GRE. Surface properties of intact cells were analysed through ATR-IR spectroscopy, and the CWPs were analysed by 2DE. The ATR-IR spectrum of *B. cereus* cells grown in different media was divided into four parts with wavelength ranging from 1000-1500 cm^-1^, 1500-2500 cm^-1^, 2500-2900 cm^-1^ and 2900-4000 cm^-1^ for a detailed analysis (Figure 2). At wavelengths 1045.2, 1256 and 1274.7 cm^-1^, *B. cereus* grown in LB, MM and MM+TRE showed bands that were not detectable in MM+GRE (Figure 2A). *B. cereus* grown in LB and MM+TRE had two distinct bands at 1650 and 1660.4 cm^-1^. Whereas, no such bands were detected when the cells were grown in MM+GRE (Figure 2B). The ATR-IR profile of *B. cereus* grown in MM+GRE in 2500-2900 cm^-1^ range was different from LB, MM and MM+ TRE, with two extra bands at 2510 and 2750 cm^-1^ (Figure 2C). In LB, MM and MM+TRE grown *B. cereus*, two split bands at 2985.3 and 3008.4 cm^-1^ were detected (Figure 2D), but in MM+GRE grown cells such bands were not detectable (Table 3). 

**Table 3:** Identification and comparative analysis of ATR-IR bands in *B. cereus* in presence.

**Wave number (cm^-1^**)**^a^**	**Media for growth of bacteria^b^**				**Function^c^**
	**LB**	**MM**	**MM+TRE**	**MM+GRE**	
1018.2	-	+	-	-	Indicate change in polysaccharide component
1045.2	+	+	+	-	C-OH bending and C-O stretching of O-cetyl ester bonds attributing to exoploymer formation
1256	+	+	+	-	
1274.7	+	+	+	-	Dipicolinic acid (invovled in sporulation) marker band for sporulating bacteria like bacilli
1635	+	+	+	+	β-sheets
1650	+	+	+	+	α-helix. Shift or split indicates stress
1660.4	+	- (shifted to 1680nm)	+	-	
2510	-	-	-	+	Hydride vibrations
2750	-	-	-	+	
2985.3	+	+	+	-	CH_3_ & CH_2_ stretching vibrations (lipids/fatty acid components of membrane/cell wall)
3008.4	+	+	+	-	

a Wavenumber (cm^-1^) at which band was detected

b Bacteria analysed after growing in different media *viz*., LB, MM, MM+TRE and MM+GRE

c Function corresponding to detected band

**Figure 3:**
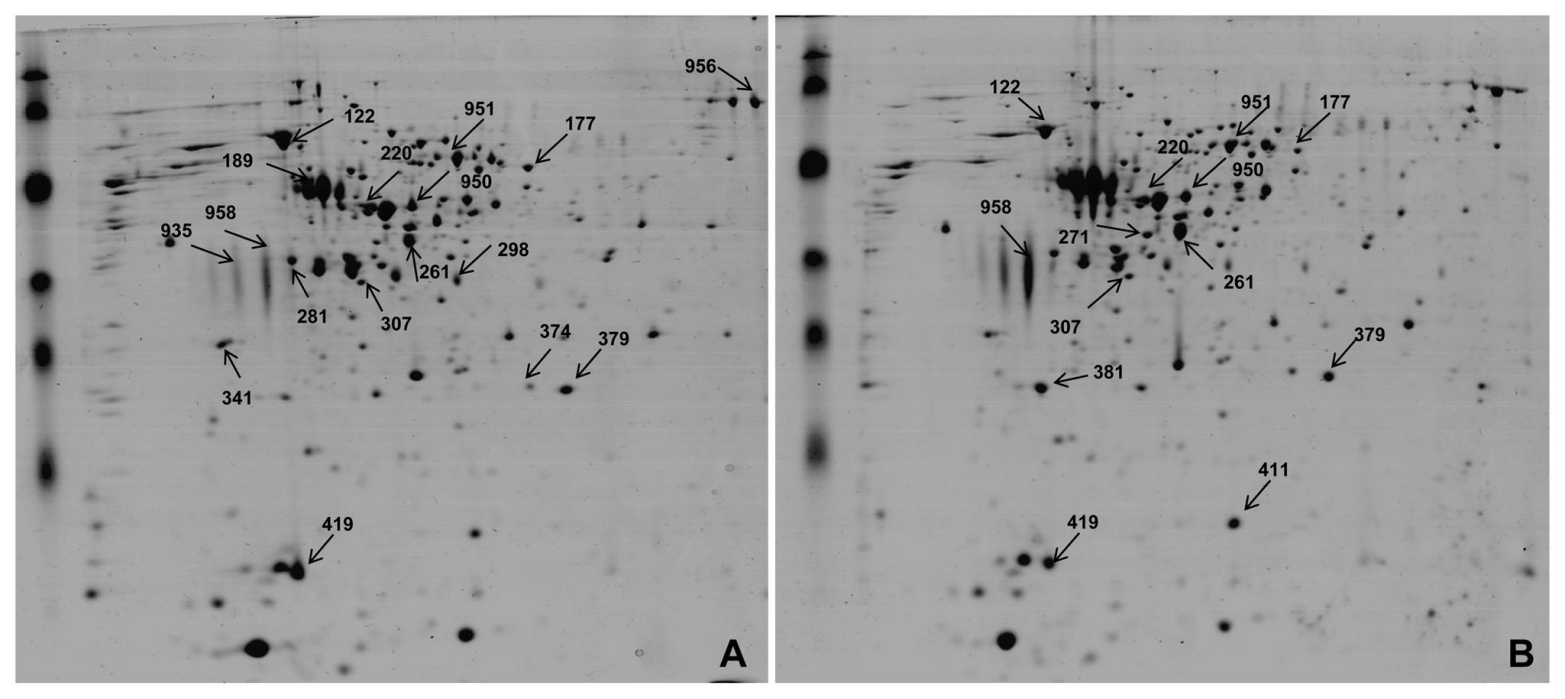
Representative 2DE gels of *B. cereus* cell-wall proteome. *B. cereus* grown in MM media amended with (A) tobacco root exudates or (B) groundnut root exudates. In the first dimension (IEF), 500 μg of protein was loaded on an 18 cm IPG strip with a linear gradient of pH 4-7 and 12.5% SDS-PAGE gels were used in the second dimension. Proteins were visualized by Coomasie blue staining. Arrows point towards the differentially expressed proteins.

*B. cereus* varied its CW-proteome upon exposure to TRE or GRE. The lactate dehydrogenase (LDH) assay showed that the CWP fraction was free from cytosolic contamination (data not shown). The CWP detected with a ratio of ≥1.5 in REs-amended MM *vs.* MM grown cells were considered as up-regulated and ≤0.5 as down-regulated. As compared to unamended control, a total of 17 and 13 CWPs were differentially expressed by *B. cereus* in TRE- and GRE-amended MM, respectively (Figure 3). In TRE-amended MM, 11 proteins were up-regulated and 6 proteins were down-regulated. In GRE-amended MM, 11 proteins were up-regulated and 2 proteins were down-regulated. Among these, 2 proteins were down-regulated and 9 were up-regulated in *B. cereus* in response to both TRE and GRE. Three proteins (Spot no. 271, 381 and 411) were significantly (p<0.05) up-regulated in CW of *B. cereus* grown in GRE-amended media as compared to that of CWP in TRE-amended MM (Figure 3). Similarly, three proteins (spot no. 341, 281 and 419) were significantly up-regulated in CW of *B. cereus* grown in presence of TRE but not in presence of GRE (Figure 3). A few of the differentially expressed proteins were mutually exclusive to TRE or GRE, and a few other proteins (Spot no. 122, 220, 177 and 951) were commonly up-regulated in response to both REs (Table 4). The up-regulated proteins like chaperonin and 2-amino-3-ketobutyrate coenzyme A ligase were expressed to a greater extent by *B. cereus* exposed to TRE than GRE (Figure 3). Identity for proteins (Spot no. 220, 271, 281, 341 and 411) could not be obtained due to lack of matching peptides.

**Table 4:** Identification and comparative analysis of differentially expressed proteins in presence of root exudates.

**Spot No.^a^**	**Fold Change (± SE)**		**Protein identification**	**Accession no. & organism^d^**	**Peptides matching**	**Score**	**Mass (kDa)/pI**
	**TRE/MM^b^**	**GRE/MM^c^**					
**Up-regulated in presence of GRE**							
381	0.69 (±0.03)	2.71 (±0.03)	Alkyl hydroperoxide reductase	gi|30018585 *Bacillus cereus* (ATCC 14579)	MLLIGTEVKPFK IEYIMIGDPTRT TITTNFNVLMEEEGLAAR	194	20.86/ 4.79
**Up-regulated in presence of TRE**							
419	2.17 (±0.09)	1.01 (±0.08)	Chain A, Dlp-2	gi|21730375 *B. anthracis*	QVANWNVLYVK FEEFYNEAGTYIDELAER LHNYHWYVTGPHFFTLHEK	337	16.51/ 4.79
**Up-regulated in presence of both TRE and GRE**							
122	2.62 (±0.03)	1.51 (±0.11)	60 kDa chaperonin	gi|160222522*B. anthracis*	SSIAQVAAISAADEEVGQLIAEAMER GFTTELDVVEGMQFDR	115	19.66/ 4.53
177	5.87 (±0.08)	3.19 (±0.05)	**2-amino-3-ketobutyrate coenzyme A ligase**	gi|30018806 *B. mycoides* Rock3-17	YGVGAGAVR HFGLSDKVDFQIGTLSK SIEILMESTELHDR	195	43.30/ 5.41
951	11.85 (±0.22)	10.01 (±0.17)	2-methyl citrate dehydratase	gi|30020418 *B.cereus* (ATCC 4579)	AHEIQGVLALENSLNR LARPLESYVMENVLFK EEIFNALSHAWIDNSSLR	132	53.76/ 5.41

a Spot no. as in Figure 3

b Fold change in TRE amended media as compared to MM

c Fold change in GRE amended media as compared to MM

Data in parenthesis indicate standard error

d Accession no. of identified protein and organism in NCBIr database

## Discussion

### Root colonization by bacteria

Rhizospheric bacteria that improve growth and increase the yield of crop plants were termed as plant growth promoting rhizobacteria (PGPR) [24]. Root colonization was linked with the efficiency of PGPR for growth improvement and disease control [25,26]. Non-rhizospheric bacteria also promote plant growth [15,27], suggesting the possibility for selection of PGPR from different habitats for seed bacterization. The present study confirmed that root colonization is a major factor for PGPR to exert the beneficial effects on plants [28]. Successful root colonization of tobacco plants by *B. cereus* increased growth compared to uninoculated plants. Failure to sufficiently colonize the roots in groundnut resulted in no change in growth. Root colonization is a competitive process influenced by host genotypes with different REs profiles [29,30], affected by characteristics of both the bacteria and the host [31] and timely response to chemical stimuli [28]. Here, we report an alteration of REs in bacterized and non-bacterized plants for both tobacco (at 20 days of growth) and groundnut (at 5 days of growth). The root colonization was different probably due to changes in the composition of REs of these two plants.

### Structural and conformational changes in CW proteins

The changes in tobacco growth-promoting *B. cereus* cells grown in different media were evident from the ATR-IR studies with respect to cell wall components (Table 3). In ATR-IR spectra, the band at 1657cm^-1^ was attributed to the amide I vibrations of structural proteins [32]. A significant difference was the split in amide I region when *B. cereus* was grown in MM+TRE media. The position of amide I absorption implies structural and conformational differences in cell proteins [33], a factor used in classifying bacteria based on IR studies. A difference in this region shows the specificity of the bacteria for specific components of REs. Similar studies were done in synthetic media using bacteria isolated from the rhizosphere of plants under different environmental conditions [34]. Most of these studies identified and characterized different bacterial strains based on the production of metabolites [35].

The splitting of the amide I band was spectroscopically detected in *Azospirillum* when grown in the presence of wheat germ agglutinin, a molecular signal in plant-microbe interactions. Bacteria showed the amide I band at about 1657 cm^-1^ under normal growth conditions, reflecting the predominance of α-helices among secondary-structure components of bacterial cellular proteins. However, the amide I band appeared to be split, with an additional band at 1632 cm^-1^ corresponding to an enhanced proportion of β-structure components [34] for cells grown under nitrogen deficiency. We detected the presence of a band at 1635 cm^-1^ for bacteria grown in all the media, whereas in the presence of enriched nutrient media (LB or TRE), the splitting occurred. This study suggests that in presence of GRE or no additional nutrients (MM alone), β-sheets were predominant. In the presence of additional nutrients, both the α-helix and β-structure of proteins were observed. Similar results were reported in *P. elgii* grown in MM showing a band at 1632.5cm^-1^ (only β-sheets) under stress, whereas in the presence of enriched nutrients (LB or TRE), the band at 1659 cm^-1^ was attributed to the presence of α-helix also [27]. Bacterial cell surface proteins were involved in stress resistance and plant root colonization [9]. Alteration in the surface proteins was linked to bacterial adaptation to the varying environmental conditions [34]. The α-helix band in IR spectra of *B. anthracis* due to the poly-glutamic acid capsule contributed to virulence [36] and to biofilm formation by non-pathogenic strains of *Bacillus* [37]. Therefore, detection of a strong α-helix band in *B. cereus* grown in TRE-amended MM suggested the adaptation of bacterial cells for tobacco root colonization. The spectroscopic changes may be related to alterations in the surface proteins of the bacterial cell and facilitate adaptation to the varying environmental conditions with special reference to nutrients exuded by the roots [34].

### Changes in exopolysaccharides and lipid-packing of CW

The bands around 1045 cm^-1^ and 1256 cm^-1^ detected in *B. cereus* grown in presence of LB, MM and MM-amended with TRE correspond to C-OH bending [38,39]. C-O stretching of O-acetyl ester bonds was attributed to exopolymer formation [13]. The band at ~1018 cm^-1^, attributed to the cellulose (or galactomannons) [40], was detected only in *B. cereus* grown in MM and not in other media, indicating a change in polysaccharide component of *B. cereus*. 

The intensity of the band at 1256 cm^-1^ in the present study, indicative of a difference in the packing of the ester groups [13], was highest in *B. cereus* cells grown in MM as compared to that in enriched media (LB) or MM+TRE suggesting weaker hydrogen bonds in the cells grown in MM. Higher intensity of band indicated formation of less number of hydrogen bonds between C=O group of lipids and polysaccharides or other chemicals which contain O-H group as predicted by Gorgulu et al. [41]. 

Prominent change in banding pattern was observed in the range of 2800-3010 cm^-1^ with the absence of two split bands of 2985.3 cm^-1^ and 3008.4 cm^-1^ in *B. cereus* grown in MM+GRE, that were present in other three media (Figure 2D). The spectral range between 2800-3010 cm^-1^ was attributed to the -CH_3_ (terminal methyl) and =CH_2_ (methylene) vibrations in the fatty acid components of membranes [35,42,43] indicating different degrees of unsaturation in acyl chains of phospholipids [44]. Similar alteration in unsaturated fatty acid content was evident through decrease of the band intensity at ~3006 cm^-1^ for *A. brasilense* grown under metal stress [42]. *B. cereus* showed variation in membrane lipid composition in response to low water activity, reduced temperature and growth in rice starch [45]. The absence of two split bands of 2985.3 cm^-1^ and 3008.4 cm^-1^ in *B. cereus* grown in GRE-amended media suggested a possible alteration in lipid-packing of cell surface, as the organism was under stress from the compounds exuded in GRE. 

### Implications of other bands

The band at 1274 cm^-1^ was identified as a marker for sporulating bacteria [13]. The effect of antimicrobial compounds on sporulating cells was also assessed by the ratio of the bands at ~1279 cm^-1^ and ~1545 cm^-1^ [36]. The 1274 cm^-1^ band was not detectable in *B. cereus* cells grown in presence of GRE, possibly due to an inhibiting effect of an unidentified compound in the GRE on sporulation of *B. cereus* affecting the survival. The bands at 2510 and 2750 cm^-1^ could not be assigned to any functional group because weak-to-moderate intensity absorption (between 2700 and 2400 cm^-1^) was not normally associated with a bonded compound [46].

### Alteration in REs profile of non-bacterized and bacterized plants

The profile of TRE and GRE differed with respect to organic acids, carbohydrates, polyols, alkanes and benzene derivatives. An alteration was detected in the profile of bacterized plant REs as compared to non-bacterized REs. A wider variety of organic acids was detected in GRE compared to TRE. The profile of sugars also changed in response to colonization of groundnut and tobacco by *B. cereus*. Sugars are known as major source of nutrients in the rhizosphere for the colonizing bacteria and carbohydrates exuded by roots also act as chemoattractants [47]. The REs alter the immediate environment and decide the survival of rhizosphere residents. The presence of rhizobacteria plays a key role in altering both exudation and morphology of roots [28,48–51]. Soybean roots did not exude quercetin and naringenin in presence of PGPR strain *Chryseobacterium balustinum* [52]. *A. brasilense* altered pH of the rhizosphere [53] and inoculation with *Azospirillum* may change root physiology and patterns of root exudation [54].

The effect of root exometabolites of tomato plants on the growth and antifungal activity of plant growth promoting *Pseudomonas* strains in the rhizosphere was dependant on the sugar and organic acid composition of REs [55]. The alteration in TRE might help in attraction of *B. cereus* and subsequent establishment in the roots. However, the compounds detected in TRE and GRE need to be quantitatively assessed for a better understanding of their effect on *B. cereus* growth, root colonization and plant growth promotion.

### Alteration in CWP profile in response to REs

CW proteins related to oxidative stress and metabolism were differentially regulated in *B. cereus* grown in REs-amended media. DNA protecting protein chain A (Dlp-2) belonging to ferritin superfamily, detected in CW components [56], protect DNA from oxidative damage [57] and detoxify ROS in the cell [58]. Upregulation of Dlp-2 in response to REs, suggests a putative role for Dlp2 in survival of bacteria in REs environment. Similarly, ferritin-like proteins were implicated in maintaining iron balance and establishment of infection by *Mycobacterium tuberculosis* [56]. In the present study, basal levels of Dlp 2 (Spot no. 419) in CWP of *B. cereus*, could be due to the absence of iron in MM. Similarly, increase in ferritin-like protein in CWP of *B. cereus* grown in RE-amended media could be due to the availability of iron. 

The alkyl hydroperoxide reductase (AHP) protein, one of the antioxidant enzymes expressed by bacteria under oxidative stress imposed by peroxides and RNI [59], was up-regulated in response to GRE. *Burkholderia cenocepacia* was persistent in the host with an increased level of flagellin proteins and reduced expression of AHP [60]. The loss of AHP activity did not affect the viability but correlated with insignificant reduction in oxidative stress resistance. A mutant lacking *ahp* gene of plant root colonizing *Azospirillum* strain, showed increased sensitivity to oxidative stress and impaired the ability of cells to aggregate and flocculate under nutrient-limiting conditions without affecting wheat root colonization [61]. The increase of AHP in GRE grown *B. cereus* CW suggests a possible involvement of AHP in resistance to oxidative stress. However, there was no positive effect on root colonization by *B. cereus*. Elevated expression of oxidative stress-response proteins in response to the REs indicate the nature of stress imposed by REs on bacteria. Bacteria grown in REs as the source of nutrients may be under oxidative stress due to high amount of organic acids and phenolics. *B. cereus* adapted to acidic and salt-stressed environments by up-regulating enzymes with antioxidative properties [62]. 

In the present study, 2-amino-3-ketobutyrate CoA ligase (Spot no. 177), which catalyzes the second reaction step on the main metabolic degradation pathway for threonine [63], was significantly up-regulated in presence of TRE. 2-Methylcitrate dehydratase (2MCD) (Spot no. 951) which is important for conversion of propionate to pyruvate *via* an intermediate 2-methyl citrate, and a chaperonin (spot no. 122) were up-regulated in CW of *B. cereus* grown in presence of both TRE and GRE, as compared to MM. The up-regulation of 2MCD could be due to the presence of propionic acid in REs of tobacco and groundnut. Chaperonins were found in the secretome of *B. anthracis* under host-simulated conditions [64]. The up-regulation of 60 kDa chaperonin, in presence of REs, suggests the role of chaperonin-mediated protein folding in the establishment of *B. cereus* on plant roots. 

Differentially expressed proteins of *B. cereus* cells grown in TRE- and GRE-amended MM were possibly involved in survival of the bacteria during interaction with REs. Alteration in the expression of proteins involved in biosynthetic metabolism and protein transport was reported in a PGPR strain of *Paenibacillus polymyxa* grown in presence or absence of barley [10].

## Conclusion

Bacteria isolated from non-rhizospheric habitats also promote growth of plants. The cell surface components of *B. cereus* were influenced by the REs of tobacco supporting the role of REs in establishment of plant-bacteria interaction. The REs positively contributed to the changes on the *B. cereus* cell surface to facilitate root colonization and subsequent plant growth in tobacco. Detailed investigations on the specific role of individual or abundant low molecular weight compounds in REs would allow verification of the host specificity in non-symbiotic plant-bacterial associations like plant-PGPR interactions.
